# *m*-Phenyl­enediamine

**DOI:** 10.1107/S1600536808039950

**Published:** 2008-11-29

**Authors:** Richard Betz, Peter Klüfers, Peter Mayer

**Affiliations:** a Ludwig-Maximilians Universität, Department Chemie und Biochemie, Butenandtstrasse 5–13 (Haus D), 81377 München, Germany

## Abstract

In the title compound, C_6_H_8_N_2_, there are four mol­ecules in the asymmetric unit, with each mol­ecule, including the H atoms on the N atoms of the amino groups, showing local *C*2 symmetry. In the crystal structure, all except one of the NH_2_ groups participate in N—H⋯O hydrogen bonding. The identified hydrogen bonds furnish a three-dimensional network. N—H⋯π contacts are observed with H⋯π distances ranging from 2.516 (17) to 2.815 (16) Å. No π-stacking of the aromatic rings is observed.

## Related literature

For the crystal structures of a series of *meta*-phenyl­enediamine salts derived from mineralic acids, see: Anderson *et al.* (2006 ▶).
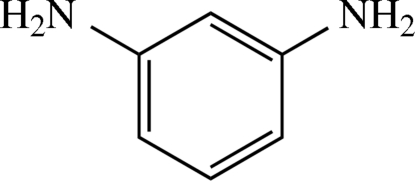


## Experimental

### 

#### Crystal data

C_6_H_8_N_2_
*M* *_r_* = 108.14
Monoclinic, 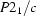

*a* = 8.1350 (4) Å
*b* = 12.0080 (6) Å
*c* = 23.9003 (16) Å
β = 90.818 (5)°
*V* = 2334.5 (2) Å^3^
*Z* = 16
Mo *K*α radiation
μ = 0.08 mm^−1^
*T* = 200 (2) K
0.32 × 0.26 × 0.22 mm

#### Data collection

Nonius KappaCCD diffractometer
Absorption correction: multi-scan (*CrysAlis RED*; Oxford Diffraction, 2005 ▶) *T*_min_ = 0.976, *T*_max_ = 0.983
13102 measured reflections
4681 independent reflections
2852 reflections with *I* > 2σ(*I*)
*R*_int_ = 0.031

#### Refinement

*R*[*F* ^2^ > 2σ(*F* ^2^)] = 0.036
*wR*(*F* ^2^) = 0.088
*S* = 0.96
4681 reflections
354 parameters
H atoms treated by a mixture of independent and constrained refinement
Δρ_max_ = 0.14 e Å^−3^
Δρ_min_ = −0.20 e Å^−3^

### 

Data collection: *CrysAlis CCD* (Oxford Diffraction, 2005 ▶); cell refinement: *CrysAlis RED* (Oxford Diffraction, 2005 ▶); data reduction: *CrysAlis RED*; program(s) used to solve structure: *SHELXS97* (Sheldrick, 2008 ▶); program(s) used to refine structure: *SHELXL97* (Sheldrick, 2008 ▶); molecular graphics: *ORTEP-3* (Farrugia, 1997 ▶); software used to prepare material for publication: *SHELXL97* and *PLATON* (Spek, 2003 ▶).

## Supplementary Material

Crystal structure: contains datablocks I, global. DOI: 10.1107/S1600536808039950/ez2150sup1.cif

Structure factors: contains datablocks I. DOI: 10.1107/S1600536808039950/ez2150Isup2.hkl

Additional supplementary materials:  crystallographic information; 3D view; checkCIF report

## Figures and Tables

**Table 1:** Hydrogen-bond geometry (Å, °)

*D*—H⋯*A*	*D*—H	H⋯*A*	*D*⋯*A*	*D*—H⋯*A*
N11—H112⋯N33^i^	0.876 (17)	2.519 (17)	3.3196 (19)	152.5 (14)
N13—H131⋯N33^ii^	0.965 (17)	2.471 (18)	3.3312 (19)	148.2 (13)
N13—H132⋯N43^iii^	0.869 (14)	2.448 (15)	3.3102 (19)	171.4 (13)
N21—H211⋯N11^ii^	0.847 (16)	2.450 (17)	3.291 (2)	172.0 (13)
N21—H212⋯N41	0.939 (16)	2.435 (17)	3.349 (2)	164.4 (14)
N23—H231⋯N21^iv^	0.876 (18)	2.574 (19)	3.443 (2)	171.3 (16)
N31—H312⋯N13^v^	0.896 (17)	2.510 (17)	3.2668 (19)	142.4 (13)
N33—H332⋯N23	0.911 (16)	2.314 (16)	3.2025 (18)	164.9 (13)
N41—H412⋯N31^vi^	0.897 (18)	2.392 (17)	3.164 (2)	144.3 (15)
N11—H111⋯*Cg*2^i^	0.914 (17)	2.573 (16)	3.4260 (14)	155.8 (14)
N23—H232⋯*Cg*1	0.887 (18)	2.516 (17)	3.2454 (15)	139.9 (15)
N31—H311⋯*Cg*4^vii^	0.922 (17)	2.815 (16)	3.7205 (16)	166.6 (13)
N33—H331⋯*Cg*2^iv^	0.860 (16)	2.608 (15)	3.2617 (13)	133.8 (13)
N41—H411⋯*Cg*1^viii^	0.868 (18)	2.707 (17)	3.5729 (15)	174.1 (16)

Symmetry codes: (i) 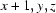; (ii) 
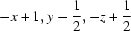
; (iii) 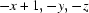; (iv) 
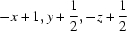
; (v) 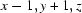; (vi) 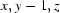; (vii) 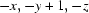; (viii) 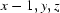. C1, *Cg*2 and *Cg*4 are the centroids of the C11–C16, C21–C26 and C41–C46 phenyl rings, respectively.

**Fig. 1.**
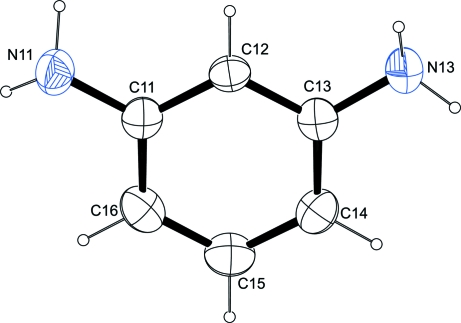
The molecular structure of the title compound, with atom labels and anisotropic displacement ellipsoids (drawn at 50% probability level) for non-H atoms.

**Fig. 2.**
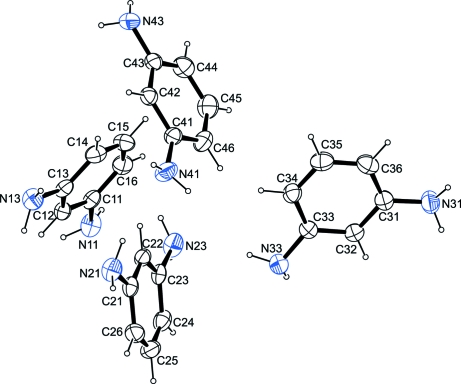
The asymmetric unit of the title compound, with atom labels and anisotropic displacement ellipsoids (drawn at 50% probability level) for non-H atoms.

**Fig. 3.**
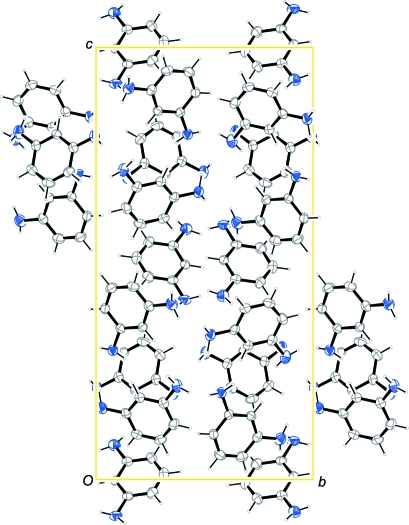
The packing of the title compound, viewed along [-1 0 0].

## supplementary crystallographic information

### Comment

In a program focused on the synthesis of derivatives of phenylarsonic acid, a number of substituted aniline-derivatives were chosen as starting materials. In order to compare the influence of an arsonic group on the geometry of some of these starting materials, the crystal structure of *meta*-phenylenediamine was elucidated by means of single-crystal X-ray diffraction.

In the molecule (Fig. 1) bond lengths and angles are normal. The H atoms on the nitrogens in each molecule are orientated towards different sides of the aromatic plane. The asymmetric unit contains four molecules (Fig. 2).

In the crystal structure, several of the amino groups participate in a classical hydrogen bonding system. In addition, N–H···π contacts are observed with H···π distances ranging from 2.516 (17)Å to 2.815 (16)Å. Details about distances and angles of these interactions are given in Table 1 (C_g_n is the centroid of the Cn1–Cn6 phenyl ring). Summarizing both these interactions, only the amino group on N43 is not involved at all.  No π-stacking of the aromatic moieties is obvious. In total, a three-dimensional network is established by these interactions (Fig. 3).

### Experimental

The compound was obtained commercially (Fluka). Crystals suitable for X-ray analysis were obtained upon slow evaporation of a solution of the compound in propan-2-ol.

### Refinement

All H atoms bonded to C atoms were calculated in idealized position and refined as riding on their parent atoms with *U*_iso_(H) values of 1.2 *U*_eq_(C). All H atoms bonded to N atoms were refined freely with individual *U*_iso_(H) values.

### Crystal data

**Table:**

C_6_H_8_N_2_	*F*_000_ = 928
*M**_r_* = 108.14	*D*_x_ = 1.231 Mg m^−^^3^
Monoclinic, *P*2_1_/*c*	Mo *K*α radiation λ = 0.71073 Å
Hall symbol: -P 2ybc	Cell parameters from 4885 reflections
*a* = 8.1350 (4) Å	θ = 3.8–26.3º
*b* = 12.0080 (6) Å	µ = 0.08 mm^−^^1^
*c* = 23.9003 (16) Å	*T* = 200 (2) K
β = 90.818 (5)º	Block, colourless
*V* = 2334.5 (2) Å^3^	0.32 × 0.26 × 0.22 mm
*Z* = 16	

### Data collection

**Table:**

Nonius KappaCCD diffractometer	4681 independent reflections
Radiation source: fine-focus sealed tube	2852 reflections with *I* > 2σ(*I*)
Monochromator: graphite	*R*_int_ = 0.031
*T* = 200(2) K	θ_max_ = 26.3º
ω scans	θ_min_ = 3.8º
Absorption correction: multi-scan(CrysAlis RED; Oxford Diffraction, 2005)	*h* = −9→10
*T*_min_ = 0.976, *T*_max_ = 0.983	*k* = −12→14
13102 measured reflections	*l* = −21→29

### Refinement

**Table:**

Refinement on *F*^2^	Secondary atom site location: difference Fourier map
Least-squares matrix: full	Hydrogen site location: difference Fourier map
*R*[*F*^2^ > 2σ(*F*^2^)] = 0.036	H atoms treated by a mixture of independent and constrained refinement
*wR*(*F*^2^) = 0.088	*w* = 1/[σ^2^(*F*_o_^2^) + (0.0454*P*)^2^] where *P* = (*F*_o_^2^ + 2*F*_c_^2^)/3
*S* = 0.96	(Δ/σ)_max_ < 0.001
4681 reflections	Δρ_max_ = 0.14 e Å^−^^3^
354 parameters	Δρ_min_ = −0.20 e Å^−^^3^
Primary atom site location: structure-invariant direct methods	Extinction correction: none

### Special details

**Table:**

Experimental. CrysAlis RED (Oxford Diffraction, 2007) Version 1.171.32.5 (release 08-05-2007 CrysAlis171 .NET) (compiled May 8 2007,13:10:02); empirical absorption correction using spherical harmonics, implemented in SCALE3 ABSPACK scaling algorithm.

### Fractional atomic coordinates and isotropic or equivalent isotropic displacement parameters (Å^2^)

**Table:**

	*x*	*y*	*z*	*U*_iso_*/*U*_eq_	
N11	1.03776 (15)	0.37351 (12)	0.21676 (6)	0.0396 (3)	
H111	1.106 (2)	0.3310 (14)	0.2386 (7)	0.066 (6)*	
H112	1.090 (2)	0.4292 (14)	0.2015 (7)	0.058 (5)*	
N13	0.77815 (16)	0.02743 (10)	0.16438 (6)	0.0382 (3)	
H131	0.7741 (19)	0.0134 (14)	0.2041 (7)	0.064 (5)*	
H132	0.6940 (17)	−0.0005 (12)	0.1465 (6)	0.043 (4)*	
N21	0.23465 (16)	0.00605 (10)	0.20759 (7)	0.0402 (3)	
H211	0.1570 (18)	−0.0254 (13)	0.2248 (6)	0.045 (5)*	
H212	0.2143 (18)	0.0130 (13)	0.1690 (7)	0.057 (5)*	
N23	0.52866 (15)	0.35624 (11)	0.19857 (7)	0.0412 (3)	
H231	0.598 (2)	0.3932 (14)	0.2199 (7)	0.065 (6)*	
H232	0.578 (2)	0.3300 (14)	0.1684 (8)	0.061 (6)*	
N31	−0.00400 (16)	0.84886 (11)	0.09377 (7)	0.0429 (3)	
H311	−0.089 (2)	0.8489 (13)	0.0677 (7)	0.062 (5)*	
H312	−0.0368 (19)	0.8796 (13)	0.1259 (7)	0.055 (5)*	
N33	0.30925 (14)	0.57749 (11)	0.20222 (5)	0.0336 (3)	
H331	0.3507 (18)	0.6315 (13)	0.2213 (7)	0.052 (5)*	
H332	0.3850 (18)	0.5226 (13)	0.1973 (6)	0.050 (5)*	
N41	0.15957 (16)	0.08484 (13)	0.07526 (6)	0.0433 (3)	
H411	0.088 (2)	0.1238 (14)	0.0935 (8)	0.065 (6)*	
H412	0.1202 (19)	0.0188 (15)	0.0637 (7)	0.065 (6)*	
N43	0.51260 (16)	0.09065 (13)	−0.08634 (5)	0.0405 (3)	
H431	0.547 (2)	0.0217 (15)	−0.0778 (7)	0.066 (6)*	
H432	0.595 (2)	0.1304 (14)	−0.1029 (7)	0.066 (6)*	
C11	0.94747 (14)	0.31141 (11)	0.17704 (6)	0.0311 (3)	
C12	0.90693 (14)	0.20145 (11)	0.18897 (6)	0.0293 (3)	
H12	0.9466	0.1683	0.2226	0.0410 (10)*	
C13	0.80913 (15)	0.13920 (11)	0.15233 (6)	0.0304 (3)	
C14	0.75103 (16)	0.18860 (12)	0.10305 (6)	0.0352 (3)	
H14	0.6827	0.1478	0.0778	0.0410 (10)*	
C15	0.79373 (17)	0.29752 (13)	0.09119 (6)	0.0393 (4)	
H15	0.7552	0.3306	0.0574	0.0410 (10)*	
C16	0.89083 (16)	0.35915 (12)	0.12736 (6)	0.0372 (4)	
H16	0.9189	0.4338	0.1184	0.0410 (10)*	
C21	0.28765 (14)	0.10438 (11)	0.23325 (6)	0.0300 (3)	
C22	0.38064 (14)	0.18093 (11)	0.20319 (6)	0.0289 (3)	
H22	0.4021	0.1678	0.1648	0.0410 (10)*	
C23	0.44212 (15)	0.27629 (11)	0.22910 (6)	0.0297 (3)	
C24	0.41196 (16)	0.29445 (12)	0.28552 (6)	0.0357 (4)	
H24	0.4552	0.3586	0.3038	0.0410 (10)*	
C25	0.31909 (16)	0.21894 (13)	0.31482 (6)	0.0405 (4)	
H25	0.2978	0.2321	0.3532	0.0410 (10)*	
C26	0.25634 (16)	0.12459 (12)	0.28946 (6)	0.0362 (4)	
H26	0.1920	0.0736	0.3103	0.0410 (10)*	
C31	0.07237 (15)	0.74448 (11)	0.09888 (6)	0.0320 (3)	
C32	0.15676 (14)	0.71468 (11)	0.14764 (6)	0.0299 (3)	
H32	0.1594	0.7644	0.1786	0.0410 (10)*	
C33	0.23748 (14)	0.61256 (11)	0.15155 (6)	0.0273 (3)	
C34	0.23643 (16)	0.54176 (11)	0.10574 (6)	0.0332 (3)	
H34	0.2934	0.4727	0.1075	0.0410 (10)*	
C35	0.15223 (17)	0.57196 (12)	0.05746 (6)	0.0409 (4)	
H35	0.1512	0.5228	0.0263	0.0410 (10)*	
C36	0.06989 (16)	0.67165 (13)	0.05351 (6)	0.0394 (4)	
H36	0.0118	0.6907	0.0201	0.0410 (10)*	
C41	0.25635 (15)	0.14386 (11)	0.03778 (6)	0.0305 (3)	
C42	0.33350 (14)	0.08948 (11)	−0.00610 (5)	0.0292 (3)	
H42	0.3138	0.0124	−0.0121	0.0410 (10)*	
C43	0.43931 (15)	0.14666 (12)	−0.04143 (6)	0.0308 (3)	
C44	0.46499 (17)	0.25939 (12)	−0.03324 (6)	0.0398 (4)	
H44	0.5361	0.2996	−0.0571	0.0410 (10)*	
C45	0.38627 (17)	0.31313 (12)	0.00996 (7)	0.0434 (4)	
H45	0.4036	0.3907	0.0153	0.0410 (10)*	
C46	0.28314 (17)	0.25683 (12)	0.04560 (6)	0.0391 (4)	
H46	0.2310	0.2952	0.0752	0.0410 (10)*	

### Atomic displacement parameters (Å^2^)

**Table:**

	*U*^11^	*U*^22^	*U*^33^	*U*^12^	*U*^13^	*U*^23^
N11	0.0309 (7)	0.0345 (8)	0.0532 (9)	−0.0038 (6)	−0.0011 (6)	−0.0040 (7)
N13	0.0419 (7)	0.0308 (8)	0.0417 (9)	−0.0045 (6)	−0.0016 (7)	−0.0037 (7)
N21	0.0367 (7)	0.0323 (8)	0.0517 (10)	−0.0029 (6)	0.0013 (7)	−0.0033 (7)
N23	0.0341 (7)	0.0324 (8)	0.0574 (10)	−0.0009 (6)	0.0063 (7)	−0.0003 (7)
N31	0.0403 (8)	0.0411 (8)	0.0473 (9)	0.0083 (6)	−0.0009 (7)	0.0068 (7)
N33	0.0347 (7)	0.0307 (7)	0.0353 (8)	−0.0010 (6)	−0.0039 (6)	−0.0004 (6)
N41	0.0437 (8)	0.0442 (9)	0.0424 (8)	0.0016 (7)	0.0161 (7)	−0.0032 (7)
N43	0.0426 (7)	0.0511 (9)	0.0278 (7)	0.0070 (7)	0.0069 (6)	0.0031 (7)
C11	0.0222 (6)	0.0340 (8)	0.0372 (8)	0.0024 (6)	0.0068 (6)	−0.0039 (7)
C12	0.0239 (6)	0.0332 (8)	0.0309 (8)	0.0042 (6)	0.0014 (6)	0.0002 (7)
C13	0.0272 (7)	0.0319 (8)	0.0322 (8)	0.0028 (6)	0.0086 (6)	−0.0046 (7)
C14	0.0352 (8)	0.0412 (9)	0.0294 (8)	−0.0012 (6)	0.0023 (6)	−0.0060 (7)
C15	0.0422 (8)	0.0478 (10)	0.0281 (8)	0.0026 (7)	0.0056 (7)	0.0066 (7)
C16	0.0355 (8)	0.0354 (9)	0.0412 (9)	−0.0011 (6)	0.0132 (7)	0.0069 (7)
C21	0.0230 (6)	0.0298 (8)	0.0370 (9)	0.0044 (6)	−0.0037 (6)	−0.0017 (7)
C22	0.0256 (7)	0.0323 (8)	0.0287 (8)	0.0061 (6)	0.0008 (6)	−0.0037 (6)
C23	0.0225 (6)	0.0281 (8)	0.0384 (9)	0.0066 (6)	−0.0010 (6)	−0.0003 (7)
C24	0.0320 (7)	0.0366 (8)	0.0385 (9)	0.0064 (6)	−0.0062 (7)	−0.0110 (7)
C25	0.0379 (8)	0.0540 (10)	0.0295 (8)	0.0126 (7)	0.0013 (7)	−0.0067 (8)
C26	0.0320 (7)	0.0414 (9)	0.0353 (9)	0.0015 (6)	0.0039 (7)	0.0060 (7)
C31	0.0259 (7)	0.0325 (8)	0.0375 (9)	−0.0032 (6)	0.0031 (6)	0.0056 (7)
C32	0.0295 (7)	0.0299 (8)	0.0305 (8)	−0.0033 (6)	0.0027 (6)	−0.0017 (6)
C33	0.0235 (6)	0.0283 (8)	0.0301 (8)	−0.0053 (5)	0.0016 (6)	0.0024 (6)
C34	0.0374 (7)	0.0265 (8)	0.0359 (9)	−0.0003 (6)	0.0031 (7)	−0.0008 (7)
C35	0.0474 (9)	0.0428 (10)	0.0325 (9)	−0.0050 (7)	0.0011 (7)	−0.0076 (7)
C36	0.0377 (8)	0.0483 (10)	0.0321 (9)	−0.0035 (7)	−0.0043 (7)	0.0037 (8)
C41	0.0256 (7)	0.0371 (9)	0.0287 (8)	0.0027 (6)	0.0017 (6)	0.0015 (7)
C42	0.0291 (7)	0.0297 (8)	0.0288 (8)	0.0013 (6)	−0.0025 (6)	0.0003 (6)
C43	0.0276 (7)	0.0393 (9)	0.0255 (8)	0.0048 (6)	−0.0027 (6)	0.0012 (7)
C44	0.0359 (8)	0.0422 (9)	0.0414 (9)	−0.0051 (7)	0.0026 (7)	0.0061 (8)
C45	0.0484 (9)	0.0297 (9)	0.0521 (10)	−0.0027 (7)	−0.0008 (8)	−0.0025 (8)
C46	0.0409 (8)	0.0346 (9)	0.0418 (9)	0.0059 (7)	0.0034 (7)	−0.0075 (7)

### Geometric parameters (Å, °)

**Table:**

N11—C11	1.4059 (18)	C15—H15	0.9500
N11—H111	0.914 (17)	C16—H16	0.9500
N11—H112	0.876 (17)	C21—C26	1.3924 (19)
N13—C13	1.3964 (18)	C21—C22	1.3960 (18)
N13—H131	0.965 (17)	C22—C23	1.3915 (18)
N13—H132	0.869 (14)	C22—H22	0.9500
N21—C21	1.3959 (18)	C23—C24	1.3913 (19)
N21—H211	0.847 (16)	C24—C25	1.378 (2)
N21—H212	0.939 (16)	C24—H24	0.9500
N23—C23	1.4016 (18)	C25—C26	1.379 (2)
N23—H231	0.876 (18)	C25—H25	0.9500
N23—H232	0.887 (18)	C26—H26	0.9500
N31—C31	1.4034 (18)	C31—C32	1.3910 (18)
N31—H311	0.922 (17)	C31—C36	1.3930 (19)
N31—H312	0.896 (17)	C32—C33	1.3935 (17)
N33—C33	1.4017 (17)	C32—H32	0.9500
N33—H331	0.860 (16)	C33—C34	1.3862 (18)
N33—H332	0.911 (16)	C34—C35	1.3818 (19)
N41—C41	1.3945 (18)	C34—H34	0.9500
N41—H411	0.868 (18)	C35—C36	1.3743 (19)
N41—H412	0.897 (18)	C35—H35	0.9500
N43—C43	1.4066 (18)	C36—H36	0.9500
N43—H431	0.895 (18)	C41—C46	1.3862 (19)
N43—H432	0.915 (18)	C41—C42	1.3925 (18)
C11—C16	1.3912 (19)	C42—C43	1.3950 (18)
C11—C12	1.3915 (18)	C42—H42	0.9500
C12—C13	1.3924 (18)	C43—C44	1.3832 (19)
C12—H12	0.9500	C44—C45	1.383 (2)
C13—C14	1.3952 (19)	C44—H44	0.9500
C14—C15	1.3837 (19)	C45—C46	1.381 (2)
C14—H14	0.9500	C45—H45	0.9500
C15—C16	1.3782 (19)	C46—H46	0.9500
			
C11—N11—H111	113.4 (10)	C24—C23—C22	119.55 (13)
C11—N11—H112	112.2 (11)	C24—C23—N23	119.61 (14)
H111—N11—H112	111.6 (15)	C22—C23—N23	120.79 (14)
C13—N13—H131	112.3 (10)	C25—C24—C23	119.67 (13)
C13—N13—H132	114.4 (10)	C25—C24—H24	120.2
H131—N13—H132	112.3 (14)	C23—C24—H24	120.2
C21—N21—H211	113.1 (10)	C24—C25—C26	121.30 (14)
C21—N21—H212	113.9 (10)	C24—C25—H25	119.4
H211—N21—H212	113.2 (14)	C26—C25—H25	119.4
C23—N23—H231	111.7 (12)	C25—C26—C21	119.70 (13)
C23—N23—H232	114.6 (11)	C25—C26—H26	120.2
H231—N23—H232	110.8 (16)	C21—C26—H26	120.2
C31—N31—H311	112.6 (10)	C32—C31—C36	119.49 (13)
C31—N31—H312	115.5 (10)	C32—C31—N31	121.00 (14)
H311—N31—H312	110.6 (14)	C36—C31—N31	119.44 (13)
C33—N33—H331	112.8 (10)	C31—C32—C33	120.59 (13)
C33—N33—H332	112.4 (10)	C31—C32—H32	119.7
H331—N33—H332	110.7 (14)	C33—C32—H32	119.7
C41—N41—H411	115.9 (11)	C34—C33—C32	119.29 (12)
C41—N41—H412	117.0 (11)	C34—C33—N33	119.73 (12)
H411—N41—H412	113.0 (15)	C32—C33—N33	120.82 (13)
C43—N43—H431	113.6 (11)	C35—C34—C33	119.73 (13)
C43—N43—H432	113.5 (11)	C35—C34—H34	120.1
H431—N43—H432	110.9 (15)	C33—C34—H34	120.1
C16—C11—C12	119.29 (13)	C36—C35—C34	121.42 (14)
C16—C11—N11	121.41 (13)	C36—C35—H35	119.3
C12—C11—N11	119.21 (13)	C34—C35—H35	119.3
C11—C12—C13	121.03 (12)	C35—C36—C31	119.45 (13)
C11—C12—H12	119.5	C35—C36—H36	120.3
C13—C12—H12	119.5	C31—C36—H36	120.3
C12—C13—C14	119.10 (13)	C46—C41—C42	119.26 (13)
C12—C13—N13	119.30 (13)	C46—C41—N41	119.99 (14)
C14—C13—N13	121.52 (13)	C42—C41—N41	120.67 (13)
C15—C14—C13	119.46 (13)	C41—C42—C43	120.91 (13)
C15—C14—H14	120.3	C41—C42—H42	119.5
C13—C14—H14	120.3	C43—C42—H42	119.5
C16—C15—C14	121.50 (13)	C44—C43—C42	119.30 (13)
C16—C15—H15	119.3	C44—C43—N43	120.73 (13)
C14—C15—H15	119.3	C42—C43—N43	119.90 (13)
C15—C16—C11	119.61 (13)	C45—C44—C43	119.45 (14)
C15—C16—H16	120.2	C45—C44—H44	120.3
C11—C16—H16	120.2	C43—C44—H44	120.3
C26—C21—N21	120.78 (14)	C46—C45—C44	121.61 (14)
C26—C21—C22	119.33 (13)	C46—C45—H45	119.2
N21—C21—C22	119.80 (13)	C44—C45—H45	119.2
C23—C22—C21	120.44 (13)	C45—C46—C41	119.45 (14)
C23—C22—H22	119.8	C45—C46—H46	120.3
C21—C22—H22	119.8	C41—C46—H46	120.3
			
C16—C11—C12—C13	−0.80 (18)	C36—C31—C32—C33	−0.47 (19)
N11—C11—C12—C13	176.01 (11)	N31—C31—C32—C33	−177.55 (12)
C11—C12—C13—C14	−0.18 (18)	C31—C32—C33—C34	1.60 (18)
C11—C12—C13—N13	176.73 (12)	C31—C32—C33—N33	−173.85 (11)
C12—C13—C14—C15	1.01 (19)	C32—C33—C34—C35	−1.62 (19)
N13—C13—C14—C15	−175.83 (12)	N33—C33—C34—C35	173.87 (12)
C13—C14—C15—C16	−0.9 (2)	C33—C34—C35—C36	0.5 (2)
C14—C15—C16—C11	−0.1 (2)	C34—C35—C36—C31	0.6 (2)
C12—C11—C16—C15	0.94 (19)	C32—C31—C36—C35	−0.6 (2)
N11—C11—C16—C15	−175.80 (12)	N31—C31—C36—C35	176.49 (12)
C26—C21—C22—C23	0.27 (18)	C46—C41—C42—C43	1.22 (18)
N21—C21—C22—C23	−176.38 (11)	N41—C41—C42—C43	−175.62 (12)
C21—C22—C23—C24	0.74 (18)	C41—C42—C43—C44	−1.28 (18)
C21—C22—C23—N23	−176.83 (11)	C41—C42—C43—N43	−178.39 (12)
C22—C23—C24—C25	−1.20 (18)	C42—C43—C44—C45	0.42 (19)
N23—C23—C24—C25	176.40 (12)	N43—C43—C44—C45	177.50 (12)
C23—C24—C25—C26	0.7 (2)	C43—C44—C45—C46	0.5 (2)
C24—C25—C26—C21	0.3 (2)	C44—C45—C46—C41	−0.5 (2)
N21—C21—C26—C25	175.81 (12)	C42—C41—C46—C45	−0.31 (19)
C22—C21—C26—C25	−0.81 (19)	N41—C41—C46—C45	176.55 (13)

### Hydrogen-bond geometry (Å, °)

**Table:**

*D*—H···*A*	*D*—H	H···*A*	*D*···*A*	*D*—H···*A*
N11—H112···N33^i^	0.876 (17)	2.519 (17)	3.3196 (19)	152.5 (14)
N13—H131···N33^ii^	0.965 (17)	2.471 (18)	3.3312 (19)	148.2 (13)
N13—H132···N43^iii^	0.869 (14)	2.448 (15)	3.3102 (19)	171.4 (13)
N21—H211···N11^ii^	0.847 (16)	2.450 (17)	3.291 (2)	172.0 (13)
N21—H212···N41	0.939 (16)	2.435 (17)	3.349 (2)	164.4 (14)
N23—H231···N21^iv^	0.876 (18)	2.574 (19)	3.443 (2)	171.3 (16)
N31—H312···N13^v^	0.896 (17)	2.510 (17)	3.2668 (19)	142.4 (13)
N33—H332···N23	0.911 (16)	2.314 (16)	3.2025 (18)	164.9 (13)
N41—H412···N31^vi^	0.897 (18)	2.392 (17)	3.164 (2)	144.3 (15)
N11—H111···Cg2^i^	0.914 (17)	2.573 (16)	3.4260 (14)	155.8 (14)
N23—H232···Cg1	0.887 (18)	2.516 (17)	3.2454 (15)	139.9 (15)
N31—H311···Cg4^vii^	0.922 (17)	2.815 (16)	3.7205 (16)	166.6 (13)
N33—H331···Cg2^iv^	0.860 (16)	2.608 (15)	3.2617 (13)	133.8 (13)
N41—H411···Cg1^viii^	0.868 (18)	2.707 (17)	3.5729 (15)	174.1 (16)

Symmetry codes: (i) *x*+1, *y*, *z*; (ii) −*x*+1, *y*−1/2, −*z*+1/2; (iii) −*x*+1, −*y*, −*z*; (iv) −*x*+1, *y*+1/2, −*z*+1/2; (v) *x*−1, *y*+1, *z*; (vi) *x*, *y*−1, *z*; (vii) −*x*, −*y*+1, −*z*; (viii) *x*−1, *y*, *z*.
